# Development of Stable *Vibrio cholerae* O1 Hikojima Type Vaccine Strains Co–Expressing the Inaba and Ogawa Lipopolysaccharide Antigens

**DOI:** 10.1371/journal.pone.0108521

**Published:** 2014-11-14

**Authors:** Stefan L. Karlsson, Elisabeth Ax, Erik Nygren, Susanne Källgård, Margareta Blomquist, Annelie Ekman, John Benktander, Jan Holmgren, Michael Lebens

**Affiliations:** 1 Department of Microbiology and Immunology at Institute of Biomedicine, The Sahlgrenska Academy at University of Gothenburg, Gothenburg, Sweden; 2 Department of Medical Biochemistry and Cell Biology at Institute of Biomedicine, University of Gothenburg, Gothenburg, Sweden; University of Padova, Medical School, Italy

## Abstract

We describe here the development of stable classical and El Tor *V. cholerae* O1 strains of the Hikojima serotype that co–express the Inaba and Ogawa antigens of O1 lipopolysaccharide (LPS). Mutation of the *wbeT* gene reduced LPS perosamine methylation and thereby gave only partial transformation into Ogawa LPS on the cell surface. The strains express approximately equal amounts of Inaba– and Ogawa–LPS antigens which are preserved after formalin–inactivation of the bacteria. Oral immunizations of both inbred and outbred mice with formalin–inactivated whole–cell vaccine preparations of these strains elicited strong intestinal IgA anti–LPS as well as serum vibriocidal antibody responses against both Inaba and Ogawa that were fully comparable to the responses induced by the licensed Dukoral vaccine. Passive protection studies in infant mice showed that immune sera raised against either of the novel Hikojima vaccine strains protected baby mice against infection with virulent strains of both serotypes. This study illustrates the power of using genetic manipulation to improve the properties of bacteria strains for use in killed whole–cell vaccines.

## Introduction

Cholera is an acute, often severe diarrheal disease which can be fatal to both children and adults [1]. It is caused in nearly all cases by *Vibrio cholerae* bacteria of a single serogroup, O1. Cholera displays a wide spectrum of symptoms from asymptomatic infection to severe dehydrating diarrhea in which patients can purge up to 2 liter of fluid per hour. Despite highly effective fluid rehydration therapies, cholera remains an important global health problem with 3–5 million cases worldwide resulting in approximately 120,000 deaths per year [2].

In many cholera–endemic countries and in cholera outbreaks following natural or political disasters clean water and basic sanitation measures are lacking and vaccines are increasingly recognized as an important preventive intervention [3]–[6]. Since 2010 the WHO recommends the use of oral cholera vaccines (OCVs) in association with other control measures both for routine preventive use in high–endemic settings and for intervention during cholera epidemics [2], [3], [7]. Currently two licensed OCVs are available both of which are killed *V. cholerae* O1 whole cell vaccines comprising a mixture of strains of Inaba and Ogawa serotypes and El Tor and classical biotypes: one (Dukoral) also contains cholera toxin B–subunit (CTB) and one (Shanchol) lacks CTB but contains a *V. cholerae* strain of the O139 serogroup (which caused geographically limited cholera outbreaks in the 1990s but has since vanished almost completely). Both vaccines have been shown in several field trials to be well tolerated and confer up to 85% protection against cholera [3], [8]–[10]. Both the Dukoral and Shanchol OCVs are relatively complex to manufacture due to their multiple strain composition. A further level of complexity results from the strains being inactivated in two different ways (heat or formalin). It would be a significant advantage if an efficacious OCV could be based on a single inactivated *V. cholerae* O1 strain co–expressing the Inaba and Ogawa serotype antigens and inactivated by one rather than two methods.

The serogroup is determined by the cell wall lipopolysaccharide (LPS), and both Ogawa and Inaba bacteria have a common O1 antigen, referred to as the A–antigen. The difference between the Ogawa and Inaba serotypes lies in the terminal perosamine of the LPS which is methylated in the Ogawa LPS forming the B–antigen, whereas the Inaba LPS is not methylated resulting in a serotype specific C–antigen [11]. The methylation resulting in the Ogawa serotype is catalyzed by an enzyme encoded by the *wbeT* gene and mutations that inactivate this enzyme such as insertions, deletions or truncation of this gene result in the Inaba serotype [12].

A third serotype called Hikojima has been described that expresses all three antigens (A, B and C), but this serotype is rare and evidence indicates that it is an unstable transitional serotype observed when a strain undergoes serotype switching from Ogawa to Inaba and does not occur in nature [13], [14] [Karlsson & Lebens, manuscript in preparation].

Recently we have worked to develop a new generation of killed OCVs based on a single, genetically modified, non–virulent strain of O1 *V. cholerae* that combines the essential components of the multiple strains used in the currently licensed vaccines. An important aspect of this work has been to generate a stable Hikojima type strain that co–expresses the Ogawa (AB) and Inaba (AC) LPS antigens. We have already reported the generation of one such strain (MS1342) achieved by the insertion into the chromosome of a parent Inaba strain of a wild–type *wbeT* gene that was stably expressed at low levels [15].

A disadvantage of this approach was the presence of two copies of the *wbeT* gene in the constructed strain with a risk for instability due to recombination. In the current paper we describe an alternative approach in which the activity of the endogenous *wbeT* gene is instead reduced by site–directed mutagenesis. Two further candidate Hikojima strains were constructed: one El Tor and one classical. We demonstrate the genetic and phenotypic stability of these novel strains as well as their ability after formalin inactivation, to elicit strong serum as well as intestinal antibody responses reacting with both Ogawa and Inaba LPS following oral route immunizations in mice.

Furthermore, passive protection studies showed that immune sera raised against either of the novel Hikojima type whole–cell vaccines protected baby mice against infection with virulent Ogawa as well as Inaba strains.

## Results

### Mutagenesis of the wbeT gene and serotype switching

**Figure 1:**
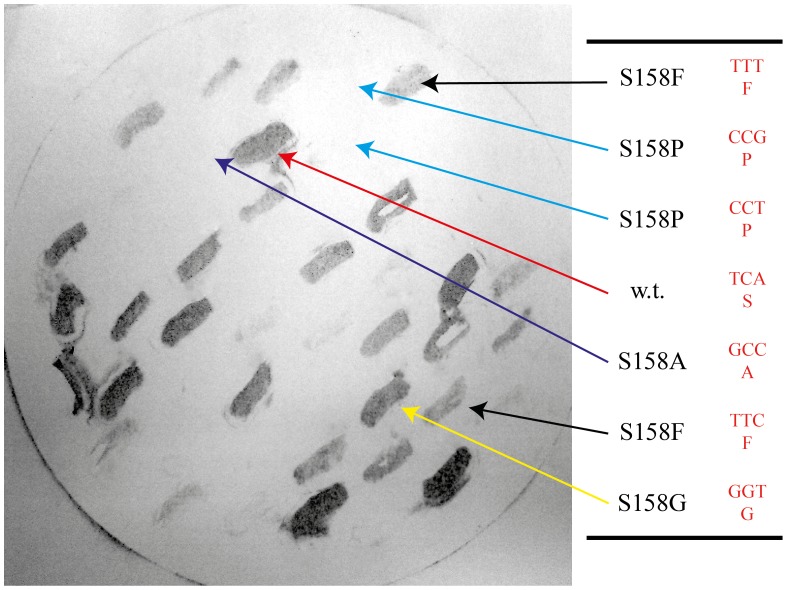
Colony blot results. O1 Inaba *V. cholerae* strain JS1569 was transformed with expression plasmids carrying different mutant *wbeT* genes. Different mutations in the *wbeT* gene give different levels of expression of the Ogawa antigen as seen by different levels of staining following labelling with Ogawa–specific antibodies. The plasmids were isolated and the *wbeT* genes sequenced. The mutations present in the different clones are indicated.

We determined that a point mutation in *wbeT* creating the mutation S158P in the gene product completely destroyed its LPS methylation activity thus resulting in the Inaba serotype. We hypothesized that this site is important for the activity of the *wbeT*–encoded protein and therefore could be used as a target site for directed mutagenesis in order to generate mutants with reduced activity of the *wbeT* gene product. A mutation library was constructed in the classical Inaba strain JS1569 as described in the Material and Methods. Fifty different colonies from the mutation library were tested using antibody–based colony blotting. It was clear that the colonies expressed different amounts of Ogawa LPS reflected in different staining intensity from nil to strong. Selected clones from the plate were further analyzed by sequencing the *wbeT* gene in their plasmids. A typical blot is shown in Figure 1 together with the sequence of selected clones. Twenty–eight additional plasmids were sequenced and besides plasmids carrying the wild–type serine the following substitutions were identified at amino acid 158; A,V,L,I,F,W,M,P,G,T,C,R, and stop codons. Interestingly, no highly polar amino acids were found at position 158 other than a single clone encoding for arginine.

**Figure 2:**
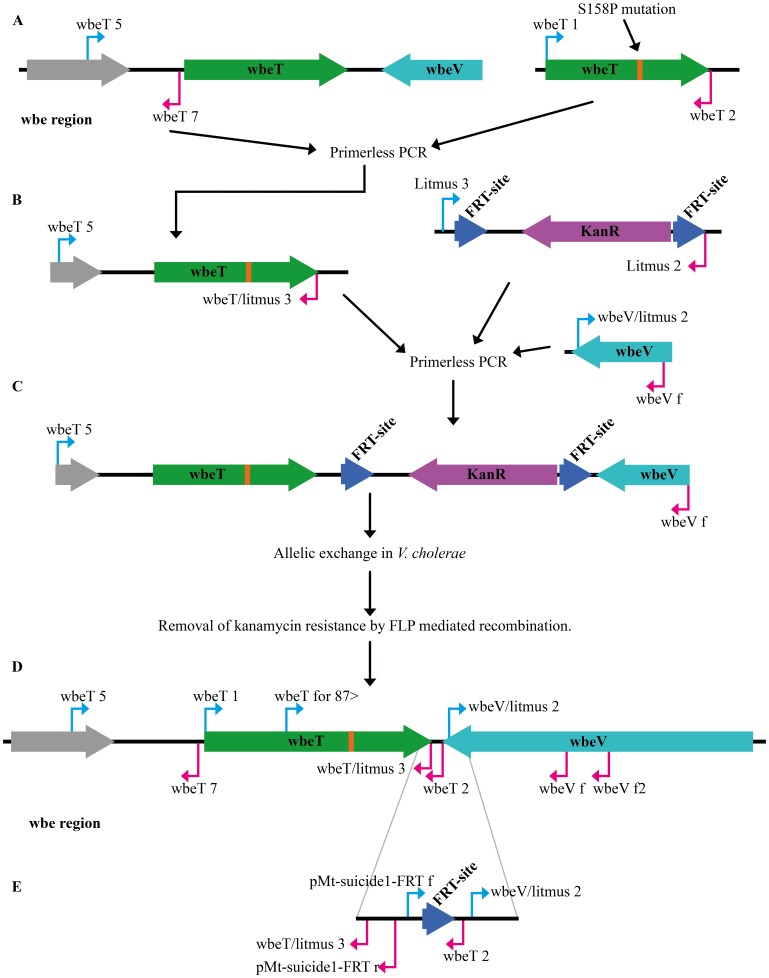
A schematic figure over the construction of the suicide plasmid and its incorporation into and it’s the removal from the genome. (A) Amplification of the mutated *wbeT* gene the upstream DNA was generated to be fused together in a primerless PCR. (B) The fused sequenced were used in another primerless PCR together with the kanamycin resistant gene flaked by FRT–sites and the downstream sequenced of *wbeT* gene. (C) The result of the second primerless PCR used to be incorporated into the pMT–suicide–sacB plasmid for integration into the genome of O1 *V. cholerae*. (D) Primer orientation of the wbe region before and (E) after gene manipulation.

Since the s158f mutant was found on the basis of comparison in dot blots with clones expressing the wild type *wbeT* gene and clones expressing inactive derivatives of *wbeT* and agglutination assays to result in activity giving the closest balance of Inaba/Ogawa expression this mutation was selected for further experiments. The *wbeT* gene carrying the s158f encoding mutation together with its native promoter was inserted into the pMT–suicide1–SacB plasmid and conjugated into the classical strain JS1569 and El Tor strain Phil6973. Colonies were selected for chloramphenicol and kanamycin resistance and positive clones were screened for the Hikojima phenotype using agglutination with serotype–specific antisera. Growth on sucrose was used to select for loss of the plasmid and kanamycin resistant strains were screened for expression of the Ogawa antigen. Finally, the kanamycin resistance gene was removed using FLP driven recombination as described in Methods. Three different strains, MS1568 Hikojima and MS1571 Ogawa from Phil6973 and MS1580 Hikojima from JS1569 were generated. The Ogawa strain had the wild type codon TCA (serine) at the position 158 whereas the Hikojima strains had TTT (Phenylalanine). Both strains from Phil6973 had a wild type codon at position 252 and the strain from JS1569 at position 219, where the respective native strains have stop codons. Both strains proved to have a stable phenotype over more than 100 generations of cultivation. The position of the PCR primers of the *wbe* region before and after gene manipulation is shown in Figure 2 D and E.

### Determination of proportions of Ogawa and Inaba antigens in the generated Hikojima strains

All vaccine candidates were tested for agglutination using serotype–specific antisera before and after formalin–inactivation as described [15], [16]. The preparations of MS1568 and MS1580 agglutinated equally well with both Ogawa and Inaba antisera, and formalin inactivation of strains preserved both the Ogawa and Inaba antigens and did not change the agglutination activity. In order to assess the relative amounts of Ogawa and Inaba antigens on the bacterial surface, inhibition ELISAs were done as previously described [15] testing the capacity of serial dilutions of formalin–inactivated test and reference Ogawa and Inaba organisms to inhibit the ELISA reactivity of antigen–specific and cross–reactive antibodies. The results confirmed that the generated MS1568 and MS1580 Hikojima strains could in a dose–dependent way inhibit each of the anti–A, anti–B or anti–C reactive antisera supporting their co–expression of substantial amounts of all of the A, B and C LPS epitopes.

However, the ca 2–fold variation of this assay did not allow for more precise estimates and in order to obtain a better quantification of the relative amounts of each antigen an ELISA–based method was developed testing the reactivity of purified LPS preparations from the Hikojima strains with Inaba– and Ogawa–specific antisera in comparison with the reactivity of the same antisera with defined mixtures of Inaba and Ogawa LPS in different proportions. Using this assay the strains MS1568 and MS1580 were estimated to have 56±4% and 42±4% (mean ± SEM of 3 tests) of their total LPS being of the Ogawa type, respectively.

**Figure 3:**
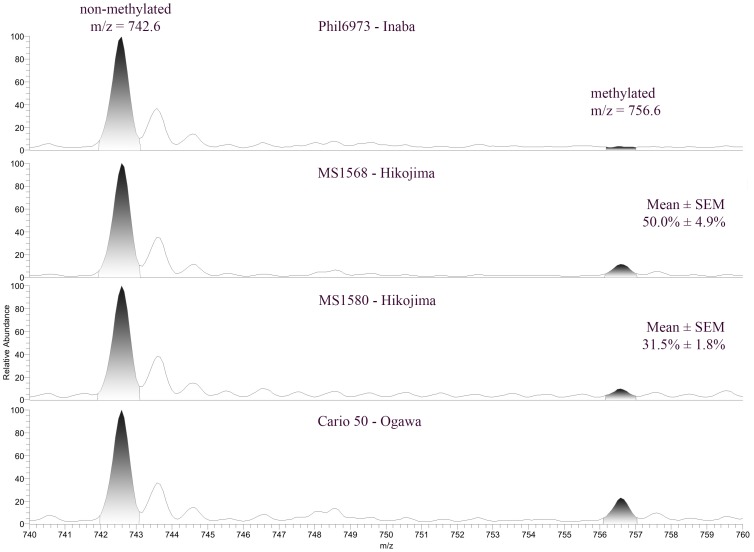
Determinations of the relative amounts of Ogawa antigen in LPS preparations of the generated Hikojima strains MS1568 and MS1580 based on amounts of methylated and un–methylated perosamine analyzed with mass spectrometry in relation to similar analyses of LPS preparations from reference Inaba Phil6973 and Ogawa Cairo 50 strains. The area under the curve for the methylated part (m/z ratio 756.6) divided by the area under the curve for the non–methylated part (m/z ratio 742.6) subtracted with the background (this ratio from the Inaba strain Phil6973) was used for the two Hikojima strain (MS1568 and MS1580) LPS preparations to calculate their percentage of Ogawa LPS (compared to the 100% Ogawa in the LPS from the Cairo50 reference strain). Diagrams are from one of 3 such experiments showing closely similar patterns and with the calculated mean ± SEM percentages Ogawa antigen indicated for MS1568 and MS1580.

In order to quantify the relative amount of Ogawa antigen using a non–immunological method, LPS preparations from different Hikojima strains and from reference Inaba and Ogawa strains were analyzed using mass spectrometry. It is known that each Ogawa LPS molecule consists of a chain of perosamine residues in which the terminal perosamine is methylated. In contrast, Inaba LPS lacks any methylated perosamine [11]. By measuring the amount of specifically methylated and non–methylated perosamine in the LPS preparations we were able to calculate the relative amount of Ogawa LPS in MS1568 and MS1580. All samples were run in triplicate and gave 50±4.9% and 31.5±1.8% for each strain respectively (presented as mean ± SEM), see Figure 3.

### Immune responses to formalin–inactivated whole–cell vaccines of the novel Hikojima strains

To evaluate the immunogenicity of inactivated whole–cell vaccine preparations of the different Hikojima–type candidate vaccine strains in comparison with the widely licensed Dukoral vaccine, both inbred Balb/c and outbred CD1 mice were immunized orally/intragastrically with equivalent amounts (based on the LPS content) of formalin–killed MS1568 or MS1580 formalin– inactivated whole–cell vaccines or with Dukoral. The formalin inactivated MS1568 vaccine and similarly prepared vaccine from its ancestor strain Phil6973, were also used for intraperitoneal immunizations. Following immunizations, serum samples were analyzed for vibriocidal antibodies against *V. cholerae* O1 Ogawa and Inaba target bacteria and by ELISA for IgG+IgM antibodies against Ogawa and Inaba LPS; similar analyses were also performed with serum samples that had been absorbed with formalin–inactivated Inaba or Ogawa bacteria to determine serotype–specific antibodies. In addition, after oral immunizations fecal pellets extracts and small intestinal tissue extracts were tested by ELISA for specific IgA antibody levels.

#### Intestinal–mucosal IgA and serum vibriocidal antibody responses after oral immunization

**Figure 4:**
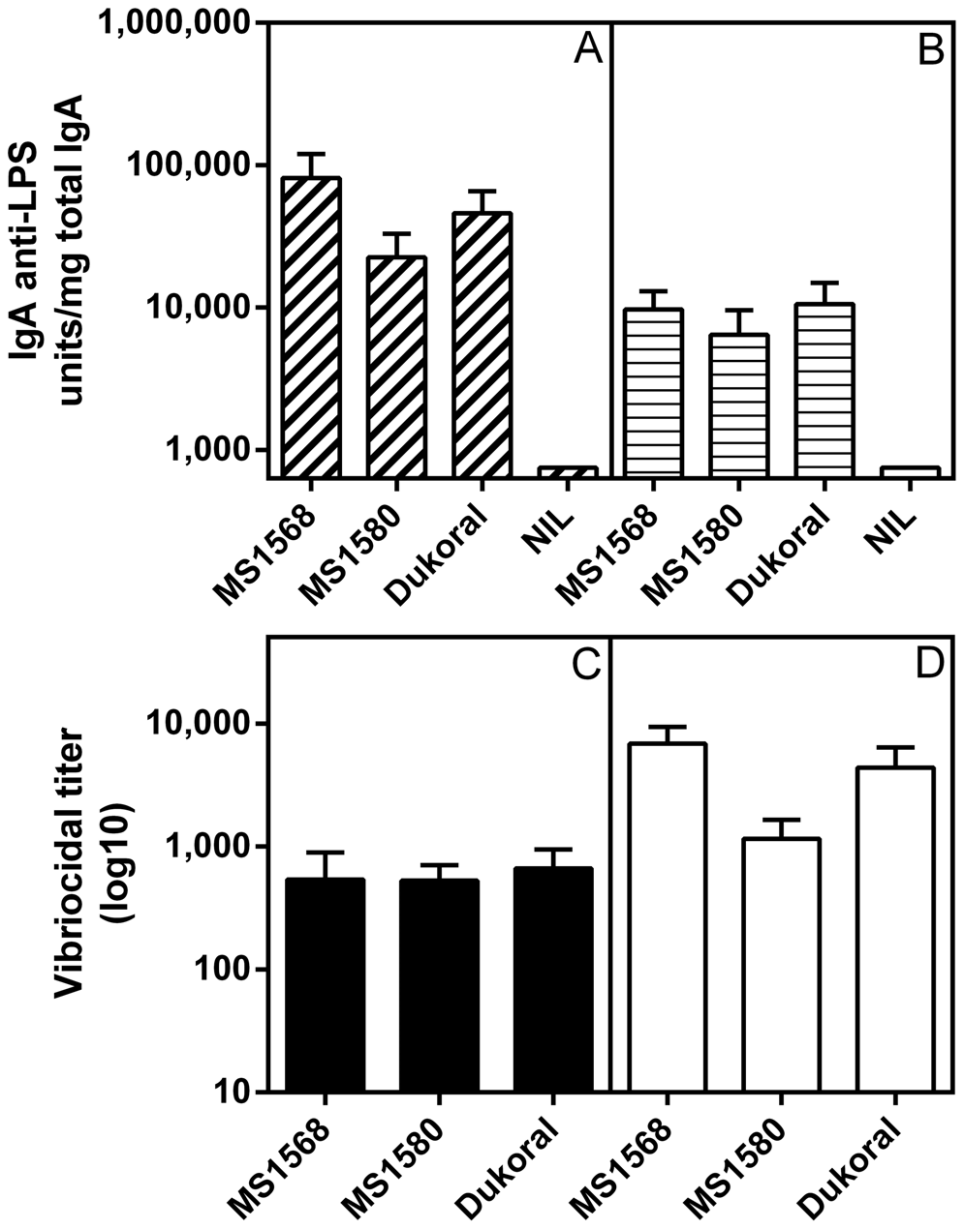
Intestinal–mucosal IgA anti–LPS and serum vibriocidal antibody responses elicited by two rounds of oral immunizations in Balb/c mice two weeks apart with formalin–killed MS1568 and MS1580 whole cell vaccines as compared to Dukoral vaccines; immunizations and collection of tissue specimens are fully described in Materials and Methods. (A) IgA anti–LPS antibody levels in fecal extracts (expressed as units per mg of total IgA measured by ELISA); and (B) the same in small intestinal tissue extracts. (C) Serum vibriocidal antibody responses against Inaba test organisms; and (D) the same against Ogawa test organisms. Bars represent the pooled results (geometric mean values ± SEM) from two separate experiments in Balb/c mice, each with 8 animals per group. Analyses of data by ANOVA showed that post–immunization antibody levels did not differ significantly between any of the immunization groups.

The intended use of a whole–cell cholera vaccine is that by immunization through the oral route it should stimulate local IgA anti–LPS antibody production in the small intestine, the immune response known to mediate most or all of the protective effect [8], [10]. We therefore compared side–by–side the IgA anti–LPS responses in fecal samples and in small intestinal extracts collected 11 days after two rounds of oral/intragastric immunization of Balb/c mice with the formalin–killed MS1568 and MS1580 vaccines or with an equivalent dose of Dukoral. All vaccines induced strong IgA antibody responses in both fecal and small–intestinal extracts at levels that did not differ significantly between the vaccines as examined by ANOVA, and which in no cases were inferior in the Hikojima–vaccinated groups compared to the Dukoral–vaccinated group (Figure 4 A and B).

**Figure 5:**
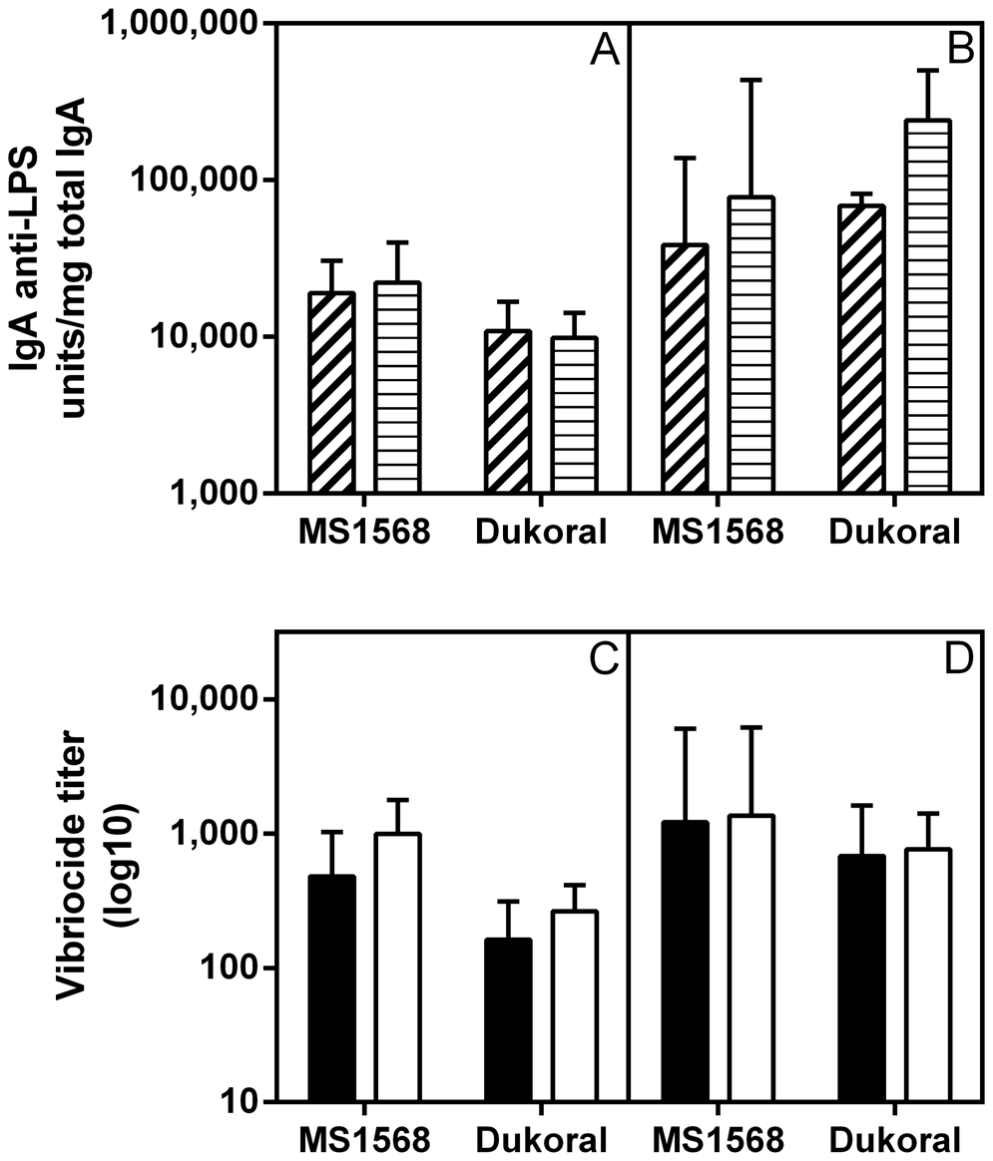
Comparison of intestinal–mucosal IgA anti–LPS and serum vibriocidal antibody responses elicited by oral immunization with formalin–killed MS1568 or Dukoral vaccines in CD1 mice. (A) IgA anti–LPS antibody levels measured by ELISA in fecal extracts (dashed) and in small intestinal tissue extracts (striped) after two rounds of intragastric immunizations and (B) same after three rounds as described in Material and Methods. (C) Serum vibriocidal antibody responses against Inaba (filled) and Ogawa (open) test organisms after two and (D) three rounds of immunizations. Bars show geometric mean values and SEM for 7 animals per group. As tested with ANOVA, post–immunization antibody levels did not differ significantly between any of the different immunization groups.

Closely similar fecal and intestinal IgA anti–LPS responses were obtained in outbred CD1 mice immunized orally/intragastrically by either two or three rounds with formalin–killed MS1568 vaccine as compared with Dukoral. The fecal and intestinal tissue extract IgA anti–LPS responses to the MS1568 vaccine were fully comparable to those against the Dukoral vaccine after either two or three rounds of immunization, which latter resulted in ca 2– to 20–fold further increased locally produced IgA levels compared to those after two immunization rounds (Figure 5A and B).

The oral immunizations with MS1568, MS1580, or Dukoral vaccines also gave rise to strong vibriocidal antibody responses (Figure 4C and D and Figure 5C and D). Again these did not differ significantly between the vaccine groups when compared by ANOVA, even though vibriocidal titers against Ogawa bacteria in sera from Balb/c mice tended to be higher after immunization with MS1568 compared to MS1580. We also measured serum IgG+IgM antibody responses to LPS by ELISA, and similar to the vibriocidal responses these responses did not differ between the immunization groups (data not shown). Also in the CD1 mice immunized orally with either MS1568 or Dukoral vaccine the vibriocidal responses to the Hikojima strain vaccine were fully comparable to that against Dukoral (Figure 5C and D), and the same was true also for IgG+IgM anti–LPS antibodies measured by ELISA (data not shown).

These results show that both the El Tor (MS1568) and the classical (MS1580) Hikojima formalin–killed whole cell vaccines were strongly immunogenic when given orally and induce intestinal IgA and serum vibriocidal and IgG+IgM anti–LPS responses that were fully comparable to those induced by the Dukoral vaccine.

#### Protective activity of immune sera after oral immunization

**Figure 6:**
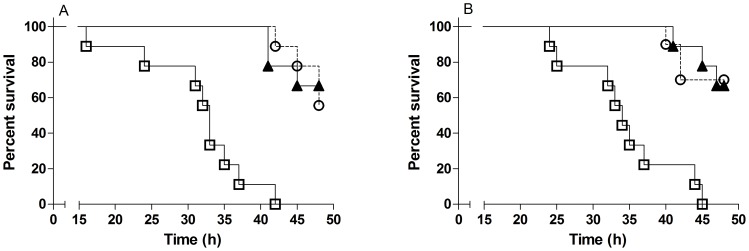
Survival curves after challenge of infant mice. Infant mice (9–10 mice per group) were challenge with Inaba (A) or Ogawa (B) bacteria mixed with pooled serum from adult CD1 mice orally immunized with formalin–killed MS1568 (open circles with dashed lines), Dukoral (triangles) or as control serum from unimmunized mice (open squares). Results show that mice challenged with either Inaba or Ogawa bacteria mixed with serum from MS1568 and Dukoral, respectively, had reduced mortality (p<0.0001) compared to mice challenged with bacteria mixed with control serum.

The protective activity of sera from CD1 mice after oral immunizations with formalin–killed MS1568 or Dukoral vaccines was tested against challenge with virulent isogenic V. cholerae O1 Inaba and Ogawa bacteria, using the infant mouse cholera model. Pooled sera from unimmunized mice served as control. The results are shown in Figure 6 and demonstrate a significant and closely similar protective activity against both Inaba and Ogawa infection by immune sera raised by the different vaccines. The results confirm the closely similar oral–mucosal immunogenicity of the Hikojima type vaccine with that of the Dukoral vaccine in mice.

#### Serum antibody responses after parenteral immunization

Titrations of vibriocidal antibodies showed that three intraperitoneal immunizations with formalin–killed preparations of MS1568 and Phil6973 gave rise to high antibody titers against both Inaba and Ogawa target strains. In the case of Phil6973 most of these antibodies were cross reactive, since absorption of the immune sera with bacteria of the heterologous serotype reduced the antibody titers against the homologous serotype more then 10–fold. The MS1568 vaccine, however, gave rise to high levels of Ogawa (anti–B) and lower levels of Inaba (anti–C) specific antibodies, the latter being produced at similar low levels by the parental Phil6973 Inaba strain, Table 1.

**Table 1:** Percentages of cross–reactive, Ogawa–specific and Inaba specific antibodies in immune sera from CD1 mice after intraperitoneal vaccination with formalin–killed bacteria of strains MS1568 and Phil6973.

Antibody specificity	Vaccine strain	
	MS1568	Phil6973
Cross–reactive	31±28%	94±2%
Ogawa–specific (anti–B)	61±28%	<0.1%
Inaba–specific (anti–C)	8±3%	6±2%

These values were calculated from vibriocidal measurements of antibody titers against Inaba and Ogawa test organisms before and after absorption of immune sera with formalin killed Inaba or Ogawa bacteria. Percentages of antibody activity shown are mean values ± SEM from 3 CD1 mice per vaccine strain.

## Discussion

In developing a new generation of killed whole cell OCVs a central aim is to make it affordable for the populations in greatest need. This can be achieved by simplifying manufacture whilst maintaining the levels of protection afforded by currently available OCVs. In current OCVs the use of several strains and two inactivation methods makes their manufacture complex and relatively expensive. Using a single vaccine strain and one inactivation method would considerably simplify and reduce the cost of manufacture.

We have previously shown that it is possible to generate stable and immunogenic Hikojima *V. cholerae* O1 derivatives co–expressing the Inaba and Ogawa O1 LPS antigens by introducing a weakly expressed wild type *wbeT* gene into an Inaba strain [15]. We now demonstrate that it is possible to generate stable Hikojima strains by substituting the inactive endogenous *wbeT* genes in Inaba strains of either El Tor or classical biotype *V. cholerae,* with a mutated *wbeT* gene giving a single amino acid substitution that reduces the activity of the gene product resulting in only partial methylation of the surface LPS.

The resulting strains MS1568 (El Tor) and MS1580 (Classical) have advantages over the previously described [15] MS1342 strain since they carry a single mutation in the *wbeT* gene and carry no genes conferring antibiotic resistance. Apart from the introduced specific mutation in the *wbeT* gene, the only change from the parental strains is the presence of a 213 base pair insert in the intergenic region between *wbeT* and *wbeV*. Both strains are potentially more stable since they carry only a single copy *wbeT* gene rather than the tandem repeat present in MS1342 and therefore the potential for recombination resulting in either Inaba or Ogawa strains is dramatically reduced.

In constructing the new strains we had to determine which mutations in the *wbeT* gene would yield the desired level of reduction in LPS methylation activity. We had identified a locus in the *wbeT* gene at which mutations had a profound effect on the activity of the *wbeT* encoded protein and employed site–directed mutagenesis in order to generate a library of mutants modifying the amino acid at position 158 of the *wbeT* gene product. Interestingly, although the mutagenesis worked well, no mutants were isolated in which highly charged residues (H,K,D,E,N) occurred at position 158. With the exception of arginine, when mutants with highly charged residues were found, they had additional mutations that introduced stop codons resulting in a truncated inactive product. This suggests that there was selection against these mutants, at least in the *E. coli* strain into which the library was transformed perhaps due to a harmful methylation activity.

Having screened the mutant *wbeT* genes and shown that they conferred differing levels of Ogawa expression, one mutation (S158F) resulting in intermediate methylation activity of O1 LPS was selected for the further work. Using a modified allelic replacement procedure the S158F mutant was used to replace the inactive mutant *wbeT* genes in two Inaba strains of O1 *V. cholerae*; one of El Tor biotype (Phil6973; a component of both the Dukoral and Shanchol vaccines) and one of classical biotype (JS1569).

The generated strains, MS1568 and MS1580 respectively, both stably expressed approximately 50% each of Ogawa and Inaba LPS as tested over more than 100 generations.

While the serotype Hikojima was originally described for a limited number of clinical isolates, recent evidence indicates that in nature Hikojima is not a stable serotype but rather represents a transition state for Ogawa to Inaba serotype switching [11]. There is only a single *wbeT* gene listed in GenBank from a Hikojima strain (Accession number FJ619106.1). This has a point mutation converting the amino acid at position 158 of the gene product from serine to proline. The same mutation is present in a number of other entries (e.g. Accession number JX565662) where the phenotype is reported as Inaba. We have found the same mutation in additional Inaba strains in our strain collection [17] and this specific *wbeT* mutation has been reported also by other groups as associated with the Inaba serotype [18], [19]. No other mutations associated with a Hikojima phenotype have been defined. We believe that to date no wild–type *V. cholerae* stably displaying the Hikojima serotype over several generations has been found.

To investigate whether the generated MS1568 and MS1580 strains were immunogenic with respect to both Inaba and Ogawa antigens we studied immune responses to these antigens in mice immunized with formalin–killed whole cell MS1568 and MS1580 vaccines and compared the responses to those elicited by immunizations with the Dukoral vaccine (which is a combined Inaba and Ogawa vaccine comprising both formalin killed and heat killed organisms). When orally immunized with formalin–killed whole–cell vaccine preparations of the generated Hikojima strains MS1568 or MS1580, all mice gave strong vibriocidal antibody responses in serum effective against Inaba as well as Ogawa test organisms as well as strong serum anti–LPS responses measured by ELISA. Both the vibriocidal and ELISA anti–LPS antibody levels induced by the Hikojima vaccines were fully comparable to those elicited by the Dukoral vaccine. Further, protection studies in infant mice showed that immune serum against the MS1568 strain after oral immunization protected to the same extent against both Ogawa and Inaba challenge and as effectively as serum obtained after similar immunization with Dukoral.

Absorption studies were used to further define the epitope specificity of the anti–LPS and vibriocidal antibodies elicited by the different vaccines. Following parenteral immunization with formalin–killed MS1568 and MS1580 bacteria both inbred Balb/c and outbred CD1 mice elicited mainly cross–reactive anti–LPS antibodies as determined by ELISA, but also significant levels of Ogawa–specific and Inaba–specific antibodies most clearly seen in the vibriocidal responses. This distribution of anti–LPS antibody specificities demonstrated that both antigens were present in an immunogenic form in the novel vaccine strains.

We attached particular importance to the ability of the candidate vaccines strains to elicit locally produced intestinal IgA antibodies to LPS, since such antibodies are regarded to be essential for immune protection after oral immunization [3], [10]. The results in both Balb/c and CD1 mice showed that IgA intestinal immune responses elicited by oral immunizations with formalin–killed MS1568 and MS1580 vaccines compared favorably with those elicited by immunizations with Dukoral with respect to IgA anti–LPS antibody responses measured by ELISA in both fecal and small intestinal tissue extracts. Of course it should be kept in mind that the strong immunogenicity of the described formalin–killed Hikojima whole–cell vaccines in mice, however promising, is no guarantee for their comparability with licensed OCVs with regard to immunogenicity and protective efficacy in humans; this can only be determined by side–by–side comparisons in humans. It could also be argued that the intragastric immunization schedule used in mice, with the vaccine dose in each round of immunization being divided for administration on consecutive days rather than as in humans given as a single administration, is not predictive of the vaccine’s future performance in humans. This schedule was chosen based on our extensive previous experience that while not changing the overall immune responses the divided doses reduce the risk of adverse effects in response to gram–negative whole–cell bacterial vaccines and also reduce the intra–group immune response variability. It is also encouraging that a similar immunization schedule and assessment of intestinal and serum antibody responses in mice to a novel oral vaccine against enterotoxigenic *E. coli* (ETEC) [20] gave results that well predicted the intestinal and serum antibody responses to the vaccine in humans [21].

Our results suggest that either of the Hikojima strains MS1568 and MS1580 could replace the mixture of strains in currently licensed OCVs. However, MS1568 may be preferable since it tended to give slightly higher antibody responses than MS1580 and is of the El Tor biotype which accounts for all cases of cholera today. It also has the advantage that it is derived from Phil6973, which is used as a formalin killed strain in both the Dukoral and Shanchol OCVs and hence has been extensively tested in humans As mentioned, a single strain and a single inactivation method should significantly simplify future manufacture of killed OCVs. Work is now in progress to develop a practical and thermo–stable OCV formulation based on formalin–inactivated MS1568 bacteria combined with a stomach acid protected recombinant cholera toxin B subunit (rCTB) that is significantly cheaper than currently licensed OCVs.

From a broader perspective this study demonstrates the value of genetically manipulated bacteria in generating killed whole cell vaccines. Unlike live attenuated vaccine strains the demands on the properties of the living bacteria are not as stringent. Although one of the generated strains (MS1580) is non–toxigenic, the second strain (MS1568) retains the toxin genes which are however not expressed under the growth conditions used for cultivation for use in the vaccine formulation.

In conclusion, the current work takes advantage of the advances made in elucidating the immunological basis of protective immunity against cholera to construct novel vaccines strains that can contribute to a novel effective and affordable cholera vaccine that is accessible to all who need it.

## Materials and Methods

### Bacterial strains and plasmids

The bacterial strains and plasmids used in the current study are shown in Table 2. All strains were maintained on Luria Bertani (LB) plates supplemented when necessary with appropriate antibiotics (ampicillin (Ap), 100 µg/ml; polymyxin B (PB), 50 µg/ml; chloramphenicol (Cm), 12.5 µg/ml; kanamycin (Km), 50 µg/ml; Rifampicin (Ra), 50 µg/ml) and stored in a 17% glycerol stock solution at –70°c. Liquid cultures were grown in LB broth at 37°c as previously described [15].

**Table 2:** Bacterial strains and plasmids used in the current study.

Bacteria		Description	Source
*E. coli*	S17–1	Tp^R^ Sm^R^ *recA, thi, pro, hsdR–M^+^* RP4∶2–Tc:Mu: Km Tn7 λpir.	[32]
	DH12s	80d*lac*ZΔM15 *mcr*A Δ(*mrr*–*hsd*RMS–*mcr*BC) *ara*D139 Δ(*ara, leu*)7697 Δ(*lac*X74 *gal*U *gal*K *rps*L (Str^R^) *nup*G *rec*A1/F′ *pro*AB^+^ *lac*I^q^ZΔ M15	Life technologies, California, USA (former Invitrogen)
*V. cholerae*	VX44945	Wild–type clinical isolate El Tor Ogawa	Matlab, Bangladesh, 1987
	T19479	Wild–type clinical isolate El Tor Inaba	Dhaka, Bangladesh, 1979
	X25049	Wild–type clinical Isolate El Tor Ogawa	Matlab, Bangladesh, 1982
	MS1489	Inaba derivative of X25049	This Study
	JS1569	Ra^R^, *ΔctxA* derivative of classical Inaba strain 569B	[33]
	MS1356	Ra^R^, Cm^R^, Ogawa derivative of JS1569 carrying the pMT–suicide1 plasmid,	[15]
	MS1580	Ra^R^, Hikojima derivative of JS1569	This Study
	Phil6973	PB^R^, Wild–type clinical isolate El Tor Inaba	India, 1973 [34]
	MS1568	PB^R^, Hikojima derivative of Phil6973	This Study
	MS1571	PB^R^, Ogawa derivative of Phil6973	This Study
	Cario 50	Wild–type clinical isolate classical Ogawa	Egypt, 1949 [35]
**Plasmids**		**Description**	**Source**
pML–wbeTtac		Expression vector carrying the *tac* promoter, the lacI^q^ repressor gene, and the wild type *wbeT* gene.	[15]
pMT–suicide1–sacB–wbeT		pMT–suicide1–sacB carrying the wild–type *wbeT* gene from VX44945 and the *sacB* gene from *B. subtilis*.	[15]
pMT–suicide1–sacB–wbeT–S158F		pMT–suicide1–sacB carrying the mutated *wbeT* gene (S158F).	This study
pML–Tn5FRT/KAN		Derivative of EZtn5 Kan2 with FRT sequences flanking the Km^R^ gene in Litmus 28	M. Lebens unpublished

### Cloning and mutagenesis of the wbeT gene

Primers and oligonucleotides used in the cloning and analysis of the *wbeT* gene are shown in Table 3. All DNA synthesis and DNA sequencing was performed by Eurofins MWG Operon, Ebersberg, Germany. Enzymes and reagents for manipulation of DNA were obtained from Thermo Fisher Scientific Inc. (Waltham, MA, USA) and reactions were carried out in buffers provided by the manufacturers according to their recommendations. Plasmids were isolated using the ZR Plasmid MiniprepTM Classic kit (Zymo Research Corp Irvine CA, USA).

**Table 3:** Primer and Oligonucleotide used for cloning and analysis of the wbeT gene.

Oligonucleotide name	DNA sequence
wbeT1	5′–CTGCATCTGCAAGTTGATTCTGTATG–′3
wbeT2	5′–ATAGTGAACTCTTCGGAAATGTCTG–′3
wbeT 5 BglII	5′–cccagatctGGCTTTAGTGAATCGCGATTTGTCGG–′3
wbeT 7	5′–CATACAGAATCAACTTGCAGATGCAG–′3
wbeT m1	5′–GGGGGTTCGAAGTTTATGAGTTTGATAATAGGGTGNNBTCATTATATTTTCA AAAAAATACAGACATAGCAGATAAGGTTAAAAATAGCCAAGTTCTGGCGCGC–′3
wbeT m3	5′–GCGCGCCAGAACTTGGCTATTTTTAACC–′3
wbeT for 87>	5′–CGGTGCAAACGTTGGAACTTTCTG–′3
wbeT/litmus 3	5′–CTGGCGTAGCTTGGCGTAATCATGGGCAATGACATACGGAATATATTAAATT ATTTTATGATAC–′3
wbeV/litmus 2	5′–CTGGCGTAATAGCGAAGAGGCCCAAGTTCAACAGACATTTCCGAAGAGTTCA CTAT–′3
Litmus 2	5′–GGGCCTCTTCGCTATTACGCCAG–′3
Litmus 3	5′–CCATGATTACGCCAAGCTACGCCAG–′3

A library of mutations of the *wbeT* gene was generated by synthesizing the oligonucleotide, wbeT m1, containing the random codon (NNB) at amino acid position 158. The oligonucleotide was mixed with the primer wbeT m3 for annealing at room temperature for 1 h. A double stranded product was generated by filling in the missing bases using T4 DNA polymerase for 5 min at room temperature in the presence of dNTPs. The enzyme was then inactivated by heating the reaction at 72°C for 10 min. The double stranded product was digested with BstBI and PflMI and ligated into the previously described [15] expression plasmid pML–wbeTtac digested with the same enzymes. The ligated DNA was electroporated into commercially obtained electro–competent cells of strain DH12s (Life technologies, California, USA). The electroporated cells (40 µl) were supplemented with 1 ml SOC medium and incubated at 37°C for 90 minutes. Five µl aliquots were spread onto LB plates supplemented with ampicillin and incubated overnight. The remainder of the cell suspension was added to 25 ml fresh LB broth supplemented with ampicillin and incubated with shaking (150 rpm) overnight at 37°C. From the overnight liquid culture aliquots were supplemented with glycerol (final concentration 17%) and stored as a library stock at –70°C. Further aliquots were used to isolate plasmid DNA representing the entire mutant library. This DNA was subsequently electroporated into the O1 Inaba *V. cholerae* strain JS1569 to create a mutation library which was used subsequently for the analysis of *wbeT* expression. The spread plates demonstrated the transformation frequency but also acted as a source of individual transformants that were subsequently analyzed by sequencing to determine the success of the mutagenesis.

### Antibody based colony blot for identification of wbeT activity

Single colonies from the mutation library created in *V. cholerae* strain JS1569 were isolated and checked for *wbeT* expression using an antibody based colony blot as previously described [22]. Briefly, an overnight liquid culture of the pML–wbeTtac–S158x –containing library was diluted and plated out to obtain single colonies. From these plates single colonies were taken at random and patched in duplicate as a matrix onto LB–Ap plates which were incubated at 37°C overnight. A nitrocellulose membrane moistened in phosphate buffered saline (PBS) was applied to the plate with the patched colonies and left for 15 min prior to being carefully lifted and transferred to blocking buffer (1% Bovine serum albumin (BSA) in PBS). The membrane was blocked for 20 minutes at room temperature before transferring to 0.1% BSA–PBS with 0.05% Tween 20 containing Ogawa specific antiserum obtained by immunization with Ogawa bacteria in rabbits and the resulting sera were extensive absorbed with Inaba bacteria as described [15]. After 2 hours incubation with gentle shaking at room temperature the membrane was washed tree times with PBS–0.05% Tween and transferred to 0.1% BSA–PBS with 0.05% Tween 20 containing anti–rabbit/HRP conjugate (Jackson Immunoresearch Europe Ltd., UK). The membrane was incubated for a further two hours, washed twice with PBS–0.05% Tween and once with PBS before developing with 4–chloro–1–naphtol and hydrogen peroxide in tris–buffered saline for 15 min.

Colonies expressing different levels of Ogawa antigen were isolated and the inserts in the pML–wbeTtac–S158x plasmids were sequenced in order to determine the amino acid present at position 158 of the cloned *wbeT* gene.

### Construction of suicide plasmid and incorporation of mutant wbeT into the genome

A scheme of the steps in the construction of the suicide plasmid used for site–directed mutagenesis of O1 *V. cholerae* strains is shown in Figure 2. Briefly, the selected *wbeT* gene was amplified from the appropriate pML–wbeTtac–S158x plasmid with the primers wbeT 1 and wbeT 2. Upstream DNA was amplified from chromosomal DNA from *V. cholerae* strain vx44945 with the primers wbeT 5 and wbeT 7 (Figure 2A). Template DNA was obtained by boiling bacterial suspensions in water as previously described [15]. The two amplified fragments were fused together using primerless PCR performed in 50 µl containing DNA template, dNTPs, buffer, and High Fidelity PCR Enzyme. The mixture was subjected to 8 cycles of amplification (94°C 30 seconds, 65°C 30 seconds, and 72°C for 1.5 minutes). The entire fragment was then amplified by addition to the reaction mixture of primers wbeT5 and wbeT litmus 3 and additional polymerase and amplifying for a further 29 cycles (94°C 30 seconds, 65°C 30 seconds, and 72°C for 1.5 minutes+2 seconds increase for each cycle). Two further fragments were generated in parallel. These were the kanamycin resistance gene from Tn5 flanked by tandem flippase recognition target (FRT)–sites from the plasmid pML–Tn5FRT/KAN (constructed in this laboratory) using primers litmus 2 and litmus 3, and a 500 bp long fragment encoding the sequence downstream of *wbeT* gene (part of the *wbeV* gene) from strain vx44945 amplified with primers wbeV litmus 2 and wbeV f. The two resulting fragments were joined to the first generated fragment by primerless pcr essentially as described above (Figure 2B). The final 3.4 kb chimeric fragment was amplified using wbeT 5 BglII and wbeV f primers (Figure 2C). The cassette was blunt end repaired using t4 DNA polymerase and ligated into pMT–Suicide1–SacB (GenBank: KF188719.1) plasmid digested with EcoRV. Ligated DNA was transformed into *E. coli* strain S17–1 and transformants were selected on the basis of their resistance to both kanamycin and chloramphenicol. The insert in plasmids carried by clones with the correct antibiotic resistance were isolated and analyzed first by restriction analysis and then by sequencing to confirm that the sequence was correct. The resulting plasmid was pMT–sucide1–SacB–wbeT(S158x)–Km.

This plasmid was then transferred into *V. cholerae* strains Phil6973 and JS1569 by transconjugation achieved by patch mating on LB plates. Cells from these matings were grown on selective plates containing Km/PB for Phil6973 and Km/Ra for JS1569. Single colonies were isolated and checked for a Hikojima phenotype by agglutination with Inaba and Ogawa–specific antisera as described before [15]. Strains with the desired double reactive phenotype were grown up in LB–Km medium for 37°C 180 rpm overnight and streaked out onto LB agar plates containing no NaCl and supplemented with sucrose to a final concentration of 6%. Single colonies from the sucrose plate were isolated and patched onto LB–Km plates and checked for kanamycin resistance (Km^R^) and chloramphenicol sensitivity (Cm^S^) as well as for a Hikojima phenotype. Cm^S^ colonies with desired phenotype were sequenced with primer wbeT 87>. A clone carrying the correct sequence was made electro competent and the flipase (FLP)–expressing plasmid 708–FLPe (Gene Bridges GmbH Heidelberg, Germany) was introduced, and bacteria grown at 30°C on LB agar plates supplemented with Cm overnight. Clones that were resistant against both chloramphenicol and kanamycin were patched onto a new LB–Cm plate and grown at 37°C overnight. Colonies with the desired Hikojima phenotype that were also Km^S^ were analyzed by PCR amplification of chromosomal DNA with the primers wbeT 5 and wbeV f2. Amplified DNA was sequenced using primer wbeT 87>.

### Lipopolysaccharide isolation

LPS was isolated from bacteria using the phenol–water extraction method as described [23]. Briefly, bacteria were grown in 500 ml LB culture overnight at 37°C with shaking and then centrifuged at 7,000×g for 15 min in pre–weighed tubes. The pellet was weighed and re–suspended in deionized water (10.25 ml/g wet bacteria) and transferred to a glass bottle with an air tight lid and mixed with 80% phenol (VWR International Ltd., Lutterworth UK) (18.75 ml/g wet bacteria) using a glass pipette. The water–phenol suspension was heated in a water bath at 65°C for 30 min, with shaking every 10 min. The phenol and the water phase were allowed to separate at 4°C overnight. The aqueous phase was transferred to a separation funnel and washed three times with chloroform purum, (Sigma Aldrich chemie GmbH, Germany). It was then dialyzed in a membrane tube MWCO 12′000–14′000, Spectra/por Dialysis Membrane (Spectrum Laboratories, Inc. CA USA), for 4 days against 5 liters of tap water at 4°C. The dialyzed solution was centrifuged at 9,000×g for 30 min and the supernatant was transferred into pre–weighed glass ampules. The ampules were frozen first for 1 hour at –4°C and then for 45 min at –70°C before they were lyophilized overnight. The ampoules were weighed and the amount of LPS was calculated. The lyophilized product was dissolved in distilled water and run on a Nu–Page Bis–Tris Mini gels (Novex, Life Technologies, California, USA) and checked for purity with Coomassie Brilliant Blue staining (Bio–Rad, California, USA) and Pierce silver stain kit (Thermo scientific, Rockford, IL, USA).

### Preparation of formalin inactivated vaccines

Three colonies from a freshly grown culture on an LB agar plate (with or without antibiotic supplementation as appropriate for different strains) were used to inoculate a starter culture of 25 ml LB medium grown at 37°C with shaking at 180 rpm overnight. Five ml of this starter culture was then used to inoculate 500 ml fresh pre–warmed LB medium which was grown at 37°C with shaking until the OD_600_ reached 1.2. The cells were then harvested by centrifugation (10,000×g for 20 min) and re–suspended in PBS to an approximate density of 2.5×10^9^ bacteria/ml. To kill the cells formaldehyde (HistoLab Products AB, Gothenburg, Sweden) was added to a final concentration of 0.02 M, and the bacterial suspension was incubated for 4 h at room temperature with shaking at 120 rpm in an airtight Erlenmeyer flask. The treated suspension was centrifuged at 10,000×g for 20 min and the resulting pellet was washed three times in PBS and then re–suspended in PBS to an OD600 of 10.0. In order to test for complete inactivation aliquots of the resulting cell suspension were spread onto horse blood agar plates and incubated at 37°C for 48 h.

### Methods for quantitation of relative amounts of Ogawa and Inabaantigens in Hikojima strains

A mass spectrometric method and two immunological assays were used to quantify the relative amounts of Ogawa and Inaba antigens in LPS preparations from Hikojima strains and in formalin–killed Hikojima vaccine formulations.

#### a) Mass spectrometric determination of proportion of methylated perosamine (Ogawa antigen) in LPS preparations

A mass spectrometric method (MS) was devised to assess the relative amount of Ogawa antigen by determining the proportions of methylated versus non–methylated perosamine in purified LPS preparations from Hikojima strains as compared to reference Inaba and Ogawa LPS preparations. We first removed the lipid A from the LPS as described in detail elsewhere [24]. Briefly, 10 mg of lyophilized LPS was dissolved in 5% acetic acid and placed at 95°C for 5 hours. The solution was then centrifuged at 12,000×g for 20 minutes and the supernatant was moved to a new tube and lyophilized. The lyophilized fraction was re–suspended in distilled water and run on a Sep–Pak QMA cartridge (Waters, Milford, MA).

### 

The resulting polysaccharide (dissolved in methanol/aceto nitrile 75∶25, by volume) was separated on a 200×0.150 mm column with 5 µm polyamine II particles (YMC Europe GMBH, Dinslaken, Germany) packed in–house, and eluted with a water gradient (A: 100% acetonitrile; B: 10 mM ammonium bicarbonate). Samples were analyzed on an LTQ linear quadrupole ion trap mass spectrometer (Thermo Electron) by LC–ESI/MS at –3.5 kV. Full–scan (m/z 500–1800, 2 microscans, maximum 100 ms, target value of 30 000) was performed, followed by data dependent MS2 scans (2 microscans, maximum 100 ms, target value of 10 000) with normalized collision energy of 35%, an isolation window of 2.5 units, an activation q = 0.25, and an activation time of 30 ms. Unmetylated perosamine (m/z ratio 742.6) and methylated perosamine (m/z ratio 756.6) peaks were localized and the respective areas under the curve for these components were calculated and used to determine the proportion of methylated to total (methylated+unmethylated) perosamine (see Figure 3). This ratio was subtracted with the background ratio for LPS from the Inaba strain Phil6973 (which should have no methylated Ogawa–specific LPS) and used to calculate the relative amount of Ogawa LPS in the two Hikojima strains (MS1568 and MS1580) compared to the similarly subtracted ratio in LPS from a reference Ogawa strain (Cairo 50) in which 100% of the LPS is of Ogawa serotype. Three separate experiments were performed with Hikojima as well as reference strain LPS preparations, and the mean ± SEM percentages of Ogawa LPS in the Hikojima strains estimated.

#### b) ELISA for determination of proportions of Ogawa antigen in LPS preparations

To determine the relative amount of Ogawa LPS antigen in the different vaccine strains purified LPS preparations of different strains were examined with Ogawa–specific (anti–B) and serotype cross–reactive (anti–A) antibodies by ELISA as described before [15]. Briefly, microtiter plates (Greiner Bio–one GmbH, Frickenhausen, Germany) were coated with the test LPS preparation and for comparisons in other wells with defined mixtures of 0–100% Ogawa (MS1356) and 100–0% Inaba (*JS1569*) purified LPS. The plates were then washed and incubated with an appropriate dilution of either anti–Ogawa (anti–B) specific polyclonal rabbit antiserum [15] or a fully serotype–cross–reactive A–epitope specific monoclonal mouse antibody (8∶4) [15], and developed with HRP–conjugated goat anti–rabbit IgG or anti–mouse IgM and OPD substrate as described [15]. The Ogawa–specific anti–B reactivities in relation to epitope A reactivity of the test LPS preparations were then compared with the reactivities of the reference LPS mixtures and used to assess the percentage of Ogawa–reactive LPS from the MS1568 and MS1580.

#### c) Inhibition ELISA for determination of bacterial cell surface LPS antigens

An inhibition ELISA performed as described before [15] was used to estimate the amounts of Ogawa and Inaba LPS antigens on the bacterial cell surface by testing the ability of serial dilutions of live and formalin killed bacterial preparations to inhibit the binding to coated Ogawa or Inaba LPS of a defined amount of Ogawa– or Inaba–specific antiserum or monoclonal antibody (8∶4) specific for the common LPS A–epitope as tested by ELISA.

### Immunizations and preparation of serum, fecal, and intestinal tissue specimens

Oral/intragastric and intraperitoneal immunizations were performed in inbred 6–8 week old female Balb/c (purchased from Taconic, Denmark) or outbred CD1 mice (Charles River Laboratories, Sweden) and collections and preparations of sera, fecal and intestinal extracts were done as described before [15] with minor modifications. For the oral immunizations the mice (7 or 8 animals per group) were immunized in two or three rounds at two week intervals, each round comprising two (CD1) or three (Balb/c) intragastric administrations on consecutive days of 100 µl formalin–killed MS1568 or MS1580 whole cell vaccines or of Dukoral mixed with 100 µl 6% (w/v) bicarbonate through a disposable feeding needle; a similarly sized unimmunized control group was also included. The Hikojima strain vaccine doses were adjusted to contain the same amount of LPS as in the Dukoral dose given (corresponding to approximately 2–3×10^9^ bacteria).

Eleven days after the last vaccine administration fecal pellets (5 from each mouse) were collected and extracts prepared [15] and stored at –80 C until tested. The animals were then sacrificed, blood collected and serum prepared. Serum aliquots were also absorbed with formalin killed cells of either JS1569 (Inaba) or MS1356 (Ogawa) as previously described [15], and total and absorbed sera were stored at –20°C until tested.

Directly after bleeding, the sacrificed mice were extensively intravenously perfused with a heparin–PBS solution to maximally remove blood from tissue, where after a defined part of the small intestine was excised and extracted with saponin as described [15]; the tissue extract was stored at –80 C until analyzed.

In addition to the orally immunized mice, other groups were immunized intraperitoneally on days 0, 8, and 18 with formalin–killed preparations of strains MS1568 and Phil6973, approximately 1×10^8^ bacteria per dose. Seven days after the last dose the animals were anesthetized with isofluoran, sacrificed, and serum samples prepared and frozen at –20°C until analyzed.

### Immunological analyses

Serum, fecal extracts, and small intestine tissue extracts were analyzed for antibodies to *V. cholerae* O1 as previously described [15], [25]. Serum samples were analyzed with vibriocidal assays and with ELISA for IgG+IgM anti–LPS antibody responses. Fecal and intestinal extracts were examined with ELISA for specific anti–LPS IgA antibody responses with titers adjusted to total IgA as described and levels expressed as ELISA units per mg of total IgA [25]. Statistical multigroup comparisons were performed using one–way ANOVA with Bonferroni’s post–test in the Prism software system GrahPad 6.04 (GraphPad Software Inc., San Dieago, CA, USA).

### The infant mouse model

The infant mouse cholera model [26], [27] was used to evaluate the protective activity of sera from groups of orally vaccinated and unvaccinated (negative control) mice against *V. cholerae* O1 Ogawa and Inaba challenge. For the challenge it was desired to use strains where the only difference was the Ogawa and Inaba serotype.

A modified method of Bhaskaran *et al* [28] was used to generate an isogenic Inaba strain of the El Tor Ogawa strain X25049 of O1 *V. cholerae*. Briefly, the strain was grown in LB broth at 37°C with shaking (180 rpm) and a log phase suspension of approximately 4×10^6^ bacteria/ml was mixed with heat inactivated absorbed polyclonal rabbit anti–Ogawa serum together with guinea pig serum (Biojet Service) as a source of complement and incubated at 37°C for 1 h with shaking. LB broth was then added and the mixture incubated for 3 h at 37°C without shaking before the mixture was plated out on blood agar plates. Single colonies were checked with serum agglutination and selected Inaba strains were sequenced with the primers wbeT 1 and wbeT 2. The strain MS1489 had a mutated *wbeT* gene and stably expressed the Inaba phenotype as tested by agglutination.

Pregnant female Swiss outbred CD1 mice were purchased from Charles River Laboratories (Sweden). Three to four days after birth infant mice weighing between 2.3 and 2.7 grams were separated from their mothers, randomly grouped in 9–10 mice per group and placed at 26°C for 6 hours before oral inoculation with virulent *V. cholerae* bacteria. The challenge experiments were performed, in principle as described previously [27], using a normally lethal dose (ca. 10^6^ colony–forming units, cfu) of LB cultured early log phase *V. cholerae* O1 bacteria pre–incubated for 30 minutes with pooled heat–inactivated immune serum from orally vaccinated or as control unvaccinated CD1 mice diluted 1∶30 in PBS. Each serum pool was tested for protective activity against both a *V. cholerae* O1 El Tor Ogawa strain (X25049) and its isogenic Inaba strain (MS1489). Inoculated infants were kept separate from dams at 26°C and carefully monitored for 48 hours, after which surviving animals were euthanized. Animals that during the course of the experiments showed clinical signs of server disease i.e. change of skin coloration from pink to blue or grey, labored breathing and persistent recumbency, were defined as moribund and immediately euthanized; but statistically defined as dead one hour later. The proportions of remaining live to total challenged mice (survival fractions) at different time points were calculated, and Kaplan–Meier type survival curves were plotted and compared using the log–rank tests; P–values were adjusted for multiple comparisons using the Bonferroni correction method [29]–[31].

### Ethics Statement

All animals were housed under specific–pathogen–free conditions and all treatments and procedures performed in accordance with the Swedish Animal Welfare Act (1988∶534) and the Animal Welfare Ordinance (1988∶539). Approval for the study was given by the Ethical Committee for Laboratory Animals in Gothenburg, Sweden (Ethical number 56/13 and 277/12). Humane endpoints were used in all animal experiments to minimize animal suffering.

Adult mice were monitored daily for the first 3 days after immunization and then at least 3 days a week during the course of each experiment. The mice were humanely sacrificed if they had more than 10% weight loss, signs of apathy, or loss of fur. The adult animals were anesthetized with isofluoran and sacrificed by cutting the pulmonary aorta. Adult mice were immunized via the non–invasive oral route or by an injection into the abdominal cavity. The adult mice were kept in covered cages with 2–10 mice in each cage with free access at all times to water, food and light. The infant mouse survival experiment had an endpoint at 48 hours post–infection; before that infected moribund animals could be identified by clinical signs of severe disease, i.e. labored breathing, persistent recumbency and change of skin coloration from pink to blue or grey and these mice were euthanized to avoid prolonged suffering. The infant mice were sacrificed by rapid decapitation using a sharp scalpel. Blood was immediately drained by placing the carcass on filter paper. Infected infant mice were monitored at least every sixth hour from 0–18 hours post–infection and at least every second hour between 18 and 48 hours post–infection. In addition to two specially trained staff from our laboratory an experienced animal caretaker was involved in the monitoring of the infant mice. The infant mice were infected via the non–invasive oral route using a non–cutting, round–tip stainless steel feeding needle. In agreement with our animal ethics permit for infant mice (227/12) no chemical agents were administered during the course of the experiments. To the best of our knowledge no publications exist that describes the use of any analgesics and anaesthetics during the course of the challenge experiments.
